# Vacuum-assisted self-expanding stents in colorectal surgery: early experiences with a novel tool

**DOI:** 10.1007/s00464-025-12116-2

**Published:** 2025-08-28

**Authors:** Michaela Ramser, Simon Nennstiel, Matteo Mueller, Christoph Schlag, Matthias Turina

**Affiliations:** 1 Department of Visceral- and Transplant Surgery, University Hospital Zurich, Rämistrasse 100, 8091 Zurich, Switzerland; 2 Department of Gastroenterology and Hepatology, University Hospital Zurich, Zurich, Switzerland

**Keywords:** Anastomosis, Anastomotic leak, VAC, Stent, Prophylactic, Therapeutic, Colorectal cancer, Colon resection

## Abstract

**Background:**

Anastomotic leakage (AL) remains a dreaded complication following colorectal resections. The routine use of diverting loop ileostomies (DLI), is associated with significant morbidity, triggering interest in alternative strategies. The VACStent Colon (VSC), a novel endoscopic treatment, combines vacuum-assisted closure with a self-expanding stent, providing luminal patency. Here we report our 1-year experience with the VSC.

**Methods:**

Retrospective analysis of patients treated with VSC at our department from 02/2024 to 03/2025 either therapeutically for AL or prophylactically in high-risk anastomoses. Outcomes, including continuous luminal patency by VSC, AL healing, complications, and need for DLI, were assessed with follow-up until endoscopic healing (EH) of AL.

**Results:**

Thirteen patients received a total of 47 VSC. Continuous luminal patency was achieved in 89.4%. Overall, 10 patients were treated with therapeutic intent (43 VSC). Nine patients achieved EH under VSC therapy (6 with DLI close, 2 awaiting DLI, one managed without DLI). One patient did not achieve EH. Three patients underwent prophylactic VSC therapy following high-risk anastomoses without a DLI. Notably, no AL occurred in this group. The most commonly observed complication was pain, particularly in distally placed VSC. Outpatient VSC therapy was successfully implemented in select cases.

**Conclusion:**

VSC shows promising results as therapeutic tool for treating colorectal AL and as prophylactic measure in high-risk anastomoses. It offers the benefits of vacuum therapy combined with mechanical stenting providing luminal patency, potentially reducing the need for DLI.

Risk factors for impaired healing of colonic or rectal anastomoses, leading to anastomotic leakage (AL), are well established [1]. Multiple studies have identified variables such as age, gender, smoking status, diabetes, obesity, pelvic radiation and other comorbidities as significant contributors to AL risk [2–4]. Notably, colorectal anastomoses, particularly those created during low anterior resections, are among the most susceptible to leakage.

A widely adopted strategy to mitigate the consequences of AL in low colorectal anastomoses is the formation of a diverting loop ileostomy (DLI) [5]. By diverting fecal content from the anastomotic site, contamination as a consequence of AL is reduced, thereby diminishing the risk and severity of pelvic sepsis [6, 7]. However, ileostomies are associated with their own spectrum of complications, including local problems like obstruction, prolapse, retraction and parastomal hernia but also systemic complications like acute kidney injury due to dehydration, electrolyte imbalances, and local complication [8, 9]. For these reasons, the routine use of DLI is increasingly being challenged [10–12].

A novel treatment modality for colorectal AL is the vacuum-assisted closure (VAC) stent [13]. This approach is adapted from the VACStent system originally developed for the upper gastrointestinal (GI) tract, which is now successfully used for different indications [14]. In 2024, the VACStent Colon (VSC) (MicroTech Europe, Dusseldorf, Germany) derivative from the original design, became available on the European market (Fig. 1). As with its upper GI counterpart, it combines the benefits of vacuum therapy—namely, clearance of the leak and promotion of granulation tissue—with the protective function of a self-expanding stent, which shields the defect from luminal contents, thereby allowing for continued oral intake and stool passage, covers the wall defect, and maintains luminal patency [15]. The VSC is deployed under endoscopic visualization and guidewire assistance.

**Fig. 1:**
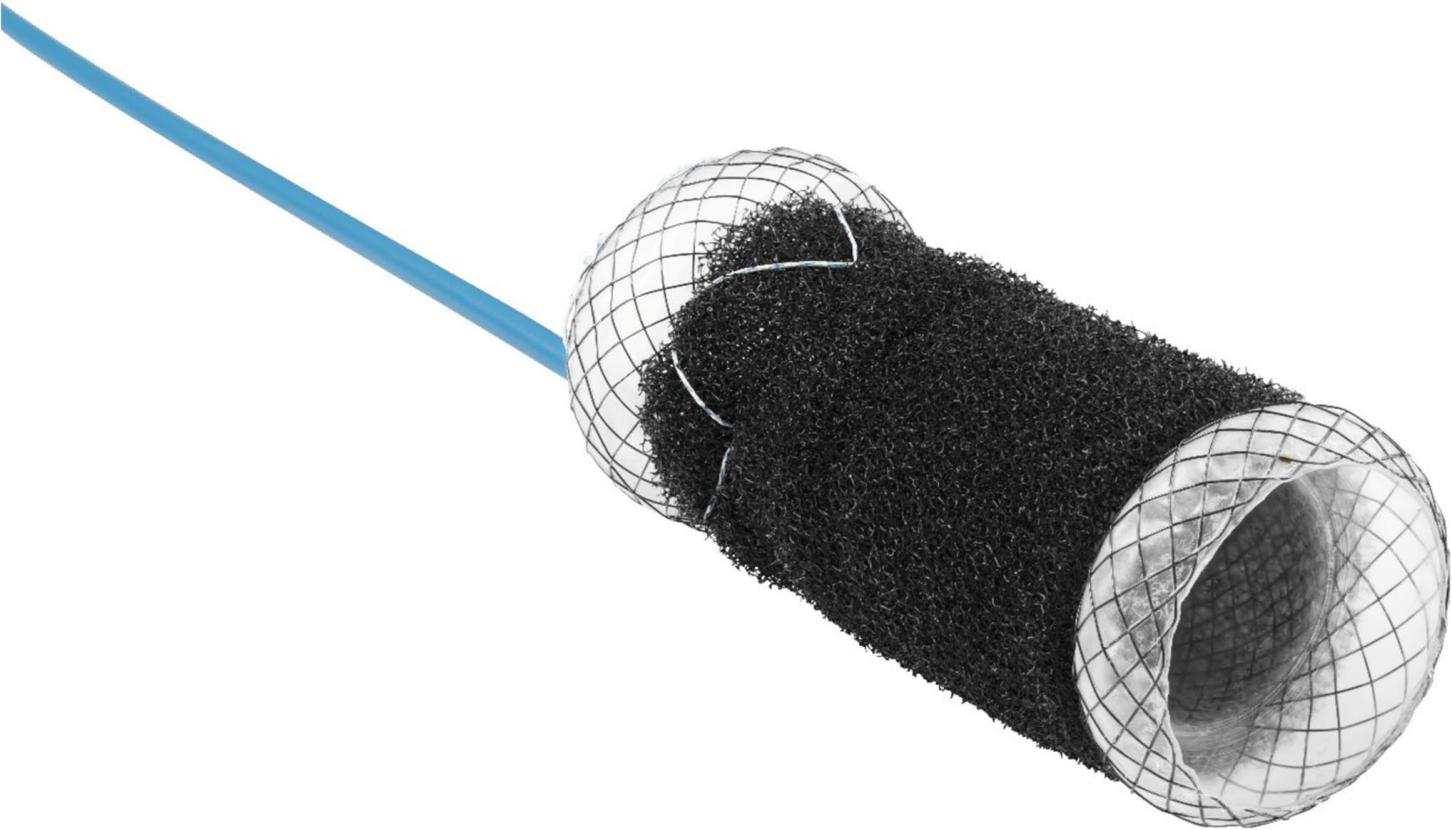
VACStent colon. Stent total length (80 mm), sponge length (60 mm), ∅ 25 mm. Image courtesy of *MICRO-TECH Europe GmbH*

The initial reports and pilot studies of the VSC for lower GI indications have demonstrated its use in both the treatment of colorectal anastomotic leaks and its prophylactic application in high-risk anastomoses, with the goal of avoiding the need for a diverting ileostomy [16].

At our institution, the first VSC was applied in 2024 [17]. Since then, its use has steadily increased for a variety of indications.

In this analysis, we present our one-year institutional experience with the VSC, both as a therapeutic intervention for colorectal AL and rectal injuries, and as a prophylactic tool to avoid DLI in the context of high-risk anastomoses. The aim is to share insights into the observed opportunities, challenges, and specific considerations.

## Methods

### Study design and setting

Retrospective observational study of patients treated with VSC at the colorectal unit in a tertiary referral center from 02/2024 to 03/2025.

Patients were identified from our prospective database on “colorectal surgery outcome quality” (BASEC 2019-00208).

The use of VSC encompassed a broad spectrum of indications, including therapeutic intervention in cases of anastomotic leakage and rectal injuries, as well as prophylactic application in patients with high-risk anastomoses, where the goal was to avoid the creation of a diverting ileostomy. Patient eligibility for VSC was determined by the responsible surgical team (M.R., M.M., and M.T.) based on clinical judgment, anatomical considerations, and the individual risk profile.

This analysis includes all cases in which VSC was deemed an appropriate treatment option following its introduction at our institution. The time period reflects one year of clinical experience, which, given the limited literature on this topic, appears to be a reasonable interval to draw initial conclusions.

Anastomotic leakages were initially suspected based on clinical signs of deviation from the expected postoperative course and were subsequently evaluated radiologically using computed tomography (CT). Final confirmation of AL was achieved through endoscopic examination. A formal classification of the size or depth of the AL was not used to describe the AL.

Each case was additionally discussed in an interdisciplinary setting with the endoscopy team to evaluate the technical feasibility and appropriateness of VSC therapy before proceeding with treatment, especially for indications that were not anastomotic leaks (AL) but for which a similar treatment approach and comparable considerations were deemed appropriate.

Comprehensive data were collected on baseline patient characteristics, specific treatment parameters, and clinical outcomes related to VSC therapy. This included complications arising during endoscopic interventions, throughout the course of inpatient care, and in case of outpatient VSC therapy, during ongoing outpatient treatment.

All complications at our department are systematically evaluated and classified according to the Clavien-Dindo grading system [18], ensuring standardized reporting of postoperative morbidity. In addition, follow-up data regarding the clinical course and therapeutic response were extracted from detailed reviews of the patients’ electronic medical records, allowing for a longitudinal assessment of VSC therapy.

### Variables

The primary outcome of the present analysis was defined as continuous luminal patency with uninterrupted negative pressure therapy, monitored through regular assessment of VSC function and endoscopic verification of device position during VSC exchanges or removal.

Secondary outcomes included the implementation of VSC therapy across different colorectal indications, specifically its use in therapeutic settings such as anastomotic leakage (AL) or rectal injury, as well as in a prophylactic context. Therapy outcomes were evaluated in terms of the treatment's ability to achieve the intended clinical goal, depending on whether the indication was therapeutic or prophylactic. In cases with therapeutic intent, the goal was endoscopically confirmed mucosal healing of the AL or rectal injury. To assess this, endoscopic follow-up was performed 6–8 weeks after completion of VSC therapy to confirm healing and enable reversal of the diverting loop ileostomy (DLI). In the prophylactic setting, treatment success was defined as the prevention of AL, verified endoscopically at the time of VSC removal.

Additionally, data were collected on VSC-related complications and the total number of VSC applications required to achieve treatment success.

### VSC treatment

The VSC was placed endoscopically either in the endoscopy suite or during surgery in the operating room. Placement was performed over a guidewire using a delivery system that deploys the stent under direct endoscopic visualization, ensuring that the anastomotic leak or rectal injury was positioned within the polyurethane sponge cylinder. The suction tube was then connected to a continuous negative pressure system applying an immediate negative pressure of − 75 mmHg. During the period of negative pressure therapy, the VSC is disconnected from the suction system once daily to allow irrigation with 20–40 mL of saline (NaCl). This routine rinse prevents excessive adhesion of the bowel wall to the sponge material.

**Fig. 2:**
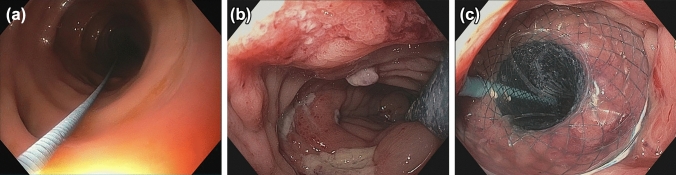
**a** Guide wire in the colon. **b** Delivery system that deploys the stent under direct endoscopic visualization. **c** VACStent Colon with blue suction tube

Typically, exchanged was performed every 5 to 7 days (Fig. 2).

Approximately six hours prior to VSC exchange, negative pressure therapy was discontinued. Following endoscopic visualization and confirmation of the VSC’s correct position, the device was removed using an endoscopic grasper. The grasper was used to pull on the distal retrieval string, causing the stent to collapse and thereby facilitating its safe extraction.

Macrogol was prescribed to patients with VSC but without proximal fecal diversion via a DLI, typically once or twice daily depending on stool consistency.

## Results

Between February 2024 and March 2025, a total of 13 patients were treated with a VSC at our institution using a total of 47 VSC. The overall mean length of therapy per stent was 5.5 days.

Mean patient age was 49.9 years, 23.1% were female. Patient baseline data and treatment details are shown in Tables 1 and 2.

**Table 1:** Baseline patient characteristics and VACStent colon indication

PatNr	Gender	Age	BMI (km/m^2^)	ASA	Diagnosis	Previous surgery	Prophylactic ileostomy	Therapeutic ileostomy	Level of anastomosis (cm)	Stapler (mm)	Indication for VACStent
#1	Male	39	34.2	2	Ulcerative colitis	Modified two stage restaurative proctocolectomy	No	No	3	31	AL IPAA
#2	Male	39	26.4	2	Upper rectal cancer	rPME		Yes	8	31	AL CR
#3	Female	43	32.8	3	Perforated diverticulitis	Sigmoid resection, ileostomy, open abdomen	Yes		15	28	AL CR
#4	Female	48	27.8	3	Rectal metastasis of melanoma	rTME, ileostomy	Yes		5	28	AL CR
#5	Male	44	26.1	2	Diverticulitis	rTME, ileostomy	Yes		5	28	AL CR
#6	Male	48	19.4	2	Transgender	Penile skin inversion		Yes	na	n.a	Rectal injury
#7	Male	55	30.4	2	Pararectal abscess	None		Yes	na	n.a	Abscess
#8	Male	21	22.5	2	Transgender	Penile skin inversion		Yes	na	n.a	Rectal injury
#9	Male	83	22.1	3	Colovesical fistula	Restauration of continuity^a^		Yes	na	n.a	AL CR, fistula
#10	Male	82	27.0	3	Sigmoid carcinoma	Sigmoid resection^a^		Yes	12	31	AL CR
#11	Male	56	29.0	4	End colostomy	Perforated diverticultis, Hartmann	No	No	14	31	Prophylactic
#12	Male	60	33.8	2	End colostomy	Perforated diverticultis, Hartmann	No	No	6	31	Prophylactic
#13	Female	32	18.4	2	Crohn colitis	Sigmoid resection	No	No	14	28	Prophylactic

*AL* anastomotic leakage, *IPAA* ileum pouch anal anastomosis, *rPME* robotic partial mesorectal excision, *rTME* robotic total mesorectal excision, *CR* colorectal, *BMI* body mass index, *ASA* American Society of Anesthesiology, *n.a.* not applicable

^a^Operation performed in other hospital

**Table 2:** VACStent Colon changes and timing

PatNr	Indication for VACStent	Initial treatment	Total No. stents	Continuous luminal patency	Duration of each VACStent (days)							Time with VACStent (days)	Outpatient VACStent	LOS (days)
					Stent1	Stent2	Stent3	Stent4	Stent5	Stent6	Stent7			
#1	AL IPAA	No other	4	4/4	5	3	3	7				18	No	37
#2	AL CR	Ileostomy, endosponge	3	2/3	4	5	7					16	No	22
#3	AL CR	OTSC, endosponge	2	2/2	6	7						13	No	48
#4	AL CR	Endosponge	3	3/3	6	7	7					20	Yes	20
#5	AL CR	No other	7	7/7	4	5^a^	5 ^a^	4	6	6	7	37	Yes	37
#6	Rectal injury	Suture	3	3/3	5	4	7					16	No	19
#7	Abscess	Ileostomy, transrectal pigtail, endosponge	2	2/2	7	7						14	Yes	14
#8	Rectal injury	Suture, ileostomy	6	4/6	3	5	4	5 ^a^	7 ^a^	7		31	No	42
#9	AL CR, fistula	Ileostomy, endosponge, nephrostomy	7	6/7	5	7	6	6	4	6	6	39	No	41
#10	AL CR	Ileostomy, drain	6	6/6	6 ^a^	4 ^a^	5 ^a^	5	7	4		31	No	32
#11	Prophylactic	n.a	1	1/1	7							7	No	19
#12	Prophylactic	n.a	2	2/2	7	5						12	No	15
#13	Prophylactic	n.a	1	0/1	6							6	No	13

*LOS* length of stay, *OTSC* over the scope clip

^a^Additional endosponge

Continuous luminal patency was achieved in 42 of 47 VSC (89.4%).

Ten patients (#1–10) were treated with VSC for colorectal anastomotic leakages, anastomotic leakage with additional involvement of the dorsal wall of the bladder with an additional colovesical fistula or anastomotic leakage after ileum pouch anal anastomosis. Indications for VSC were also rectal injuries during dissection along the anterior rectal wall during transgender surgery (penile skin inversion) or extensive pararectal abscesses of unknown origin.

Therapeutic VSC stayed for a total length of 13–37 days with a mean of 4 VSCs per patient (range 2–7) (Table 2). Therapeutic VSC were exchanged every 3–7 days, sometimes during re-look laparotomy or wound explorations which were performed every 72 h.

Three patients (#11–13) received a total of 4 VSCs in prophylactic intent, including two patients with a high-risk colorectal anastomosis following Hartmann’ reversal operations. One patient underwent sigmoid resection for segmental Crohn’s colitis. In all these three patients, a DLI could be avoided.

Prophylactic VSC were left in place for 6–12 days (one patient with two stents; the first was left in place 7 days, the second 5 days).

Treatment associated complications are listed in Table 3. The most frequently observed problem associated with VSC was pain, ranging from VAS 1–10. Dislocation CDC grade 1 are small mitigations of VSC only observed during re-endoscopy. One VSC dislocation needed an additional endoscopy to remove and place a new VSC (CDC 3a). One CDC grade 3a bleeding was treated endoscopically.

**Table 3:** VACStent treatment and associated complications

PatNr	Time (days) after VSC to stoma closure	VSC associated complications (CDC)	VSC associated complications (CDC)
#1	n.a	Bleeding (CDC 3a)	Pain (CDC 2)
#2	93	Pain (CDC 2)	Dislocation (CDC 3a)
#3	192		
#4	Pending	Pain (CDC 2)	
#5	68	Pain (CDC 2)	
#6	42	Pain (CDC 2)	
#7	525		
#8	Pending	Pain (CDC 2)	Dislocation (CDC 1)
#9	52^a^	Dislocation (CDC 1)	
#10	112		
#11	n.a		
#12	n.a	Pain (CDC 2)	
#13	n.a	Dislocation (CDC 1)	

*n.a.* not applicable, *CDC* Clavien–Dindo classification, *VSC* VAC Stent Colon

^a^Anstomotic take down and formation of colostomy

### Summary of treated patients (therapeutic) (Tables 1 and 2)

Patient #1 (therapeutic; male, 39; ulcerative colitis): AL of ileum pouch anal anastomosis (IPAA). Due to morbid obesity, it was anatomically difficult to exteriorize a loop of small bowel as DLI following fashioning of the IPAA anastomosis. After a total of 4 VSC (total of 18 days), the AL was healed endoscopically and under radiographic control. However, chronic posterior leakage developed several weeks later, successfully treated by endoscopic needle knife sinusotomy. This patient fully recovered and developed normal ileoanal pouch function.

Patient #2 (therapeutic; male, 39; rectal cancer): AL after robotic partial mesorectal excision (rPME) for a high rectal adenocarcinoma following neoadjuvant therapy with Dostarlimab. After AL diagnosis, laparoscopic lavage with intraabdominal drain placement was performed and a DLI formed. Following 3 VSC (16 days) the anastomosis healed uneventfully and the ileostomy was closed 3 months after removal of the last VSC treatment.

Patient #3 (therapeutic; female, 43; diverticulitis): AL after emergency sigmoid resection with anastomosis and DLI for perforated sigmoid diverticulitis in a patient with immunosuppression for a dermatomyositis. Due to sepsis, open abdomen treatment was done. An AL was diagnosed on the 6th postoperative day. AL was initially treated by endoscopic sponge therapy (*Braun*, Melsungen, Germany). Later, 2 VSC finalized successful anastomotic healing. The ileostomy was closed 6 months after the last VSC treatment.

Patient #4 (therapeutic; female, 48; rectal metastasis of melanoma): AL after robotic total mesorectal excision (rTME) with anastomosis and DLI for a large rectal metastasis of a malignant melanoma. The patient was under immunotherapy for the melanoma. Seven days postoperatively, AL was diagnosed and treated with 3 VSC. The anastomosis subsequently healed, which was confirmed endoscopically and radiologically. Ileostomy closure was postponed due to further chemo/immunotherapy for a new recurrence of the melanoma.

**Fig. 3:**
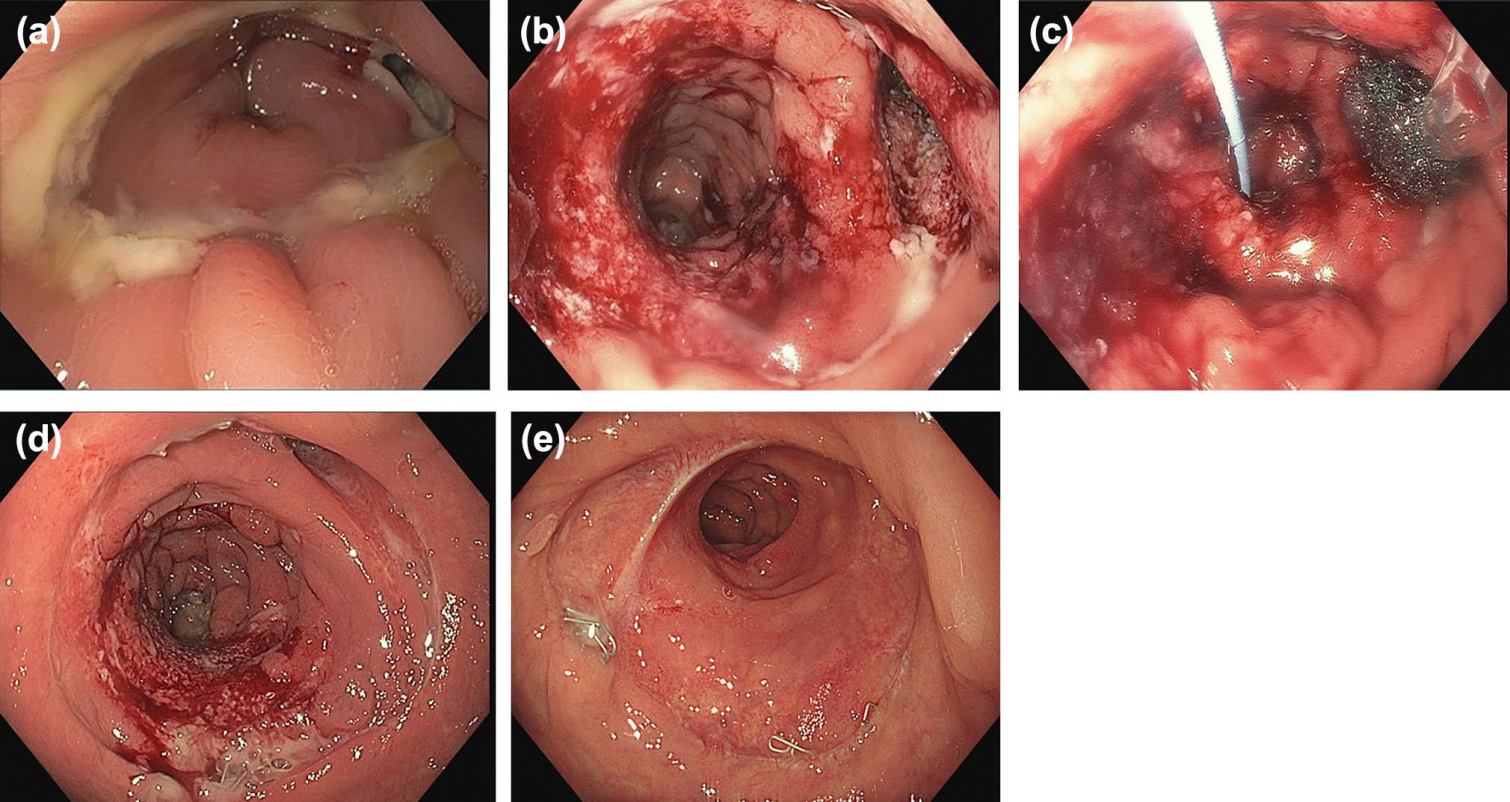
Therapeutic effect. **a** Day 1, start of VSC treatment. **b** Day 10. **c** Day 15 Placement of endosponge in cavity. Additional guide wire for VSC placement. **d** Day 25 Progressive granulation of the leakage cavity. **e** Endoscopic follow-up before ileostomy closure (week 11)

Patient #5 (therapeutic; male, 44; diverticulitis): AL after robotic sigmoid resection with was extended to a low anterior resection with total mesorectal excision for a covered perforation in the setting of an unclear inflammatory process of the sigmoid not responding to prolonged antibiotic treatment. Intraoperatively, the rectum was found to be severely inflamed and distorted by descending abscess formations, rendering a primary anastomosis unfeasible in the edematous and compromised tissue of the upper and mid rectum. A colorectal anastomosis was formed and a DLI was fashioned. Unfortunately, on postoperative day x, an AL was diagnosed radiologically and confirmed in endoscopy. The large leakage cavity was managed with endosponge (*Braun*, Melsungen, Germany) which was additionally covered with a VSC (Fig. 3). VSC use was associated with severe pain which required intermittent opioid treatment. Nevertheless, mobilisation and outpatient treatment were subsequently possible. After a total of 7 VSC, the AL was healed which was confirmed endoscopically and the ileostomy was closed 68 days following VSC removal.

Patient #6 (therapeutic; male, 48; rectal injury): A recto-neovaginal fistula was diagnosed 11 days following penile skin inversion surgery as part of gender-affirming surgery**.** The rectal defect was initially closed with sutures and diverted with a DLI for protection. Due to failure of the primary suture repair, adjunctive therapy using a VSC was initiated. After 3 VSC applications, complete healing of the rectal defect and fistula was achieved. The DLI was subsequently closed 42 days following cessation of VSC treatment.

Patient #7 (therapeutic; male, 55; pararectal abscesses): This patient presented with large pararectal and supralevatoric abscesses without rectocutaneous fistula. The abscesses were initially drained endosonography-guided transrectally with insertion of drains. To divert fecal content, an ileostomy was made and the pararectal abscesses drained with a larger transrectal surgical incision. The cavity was first treated with an endosponge. To enhance drainage and healing, a VSC was deployed as adjunctive therapy during in the follow-up endoscopy. After 2 VSC inflammation resolved completely. At follow-up, MRI demonstrated complete resolution of the collections. Interval endoscopy confirmed full mucosal healing, and the diverting ileostomy (DLI) was successfully closed X days after completion of VSC therapy.

Patient #8 (therapeutic; 21, male; rectal injury): Intraoperative lesion of the lower rectum during male-to-female gender-reassignment surgery. Suture of the tangential rectal lesion was inadequate and VSC therapy with additional DLI was started. Pain was a recurrent issue, mainly due to the very distal rectal lesion. Currently, the rectal injury has healed on endoscopic follow-up but not yet the perineal body. For this reason, DLI closure is currently postponed.

Patient #9 (therapeutic; male, 82; colovesical fistula): The patient was transferred to our unit following Hartmann’ reversal surgery at an outside hospital, initial surgery performed for perforated diverticulitis. An AL was diagnosed and treated with open abdomen treatment, repeated sutures of the anastomosis and now a remaining large colovesical fistula. Nephrostomies were placed to divert the urine and the colonic tissue around the fistula opening was treated with VSC. After 3 VSC, the fistula was suture closed transanally and the suture covered with a VSC. Endoscopy one week later showed a small remaining leakage, so that another transanal suture was placed. Again, a week later was a very small remaining leakage diagnosed with was sutured endoscopically (Apollo Overstitch, *Boston Scientific*, Malborough, MA, USA). The last VSC was in place for the next 5 days. Unfortunately, follow-up endoscopy revealed that the fistulous opening towards the bladder had reopened. As all conservative and endoscopic measures failed to show signs of healing, takedown of the anastomosis was recommended, and a definitive end colostomy was formed.

Patient #10 (therapeutic; male, 82; colon cancer): The patient was transferred to our unit after a sigmoid cancer was resected laparoscopically at an outside hospital. When AL was diagnosed, an abdominal drain was placed laparoscopically and a DLI was fashioned. When the patient arrived at our unit, endoscopy revealed a small persisting AL which was treated with VSC. Additionally, an endosponge was placed in the AL cavity. When the cavity showed good granulous tissue, the treatment was continued with VSC alone. Currently, VSC therapy is terminated with a healed anastomosis which was confirmed endoscopically 6 weeks later. The DLI closure was uneventful.

### Summary of prophylactically treated patients (Tables 1 and 2)

Patient #11 (prophylactic; male, 56; restoration of continuity after Hartmann’s): Prophylactic VSC placement in a colorectal anastomosis for a Hartmann reversal in a permanently anticoagulated patient on a ventricular assist device awaiting heart transplantation. The VSC was removed after 7 days, revealing a completely healed anastomosis.

Patient #12 (prophylactic; male, 60; restoration of continuity after Hartmann’s): The patient was referred to our unit for Hartmann’s reversal following a leak of the colorectal anastomosis after sigmoid resection for diverticulitis. Initial endosponge therapy had resulted in a significant anastomotic stricture, necessitating takedown of the original anastomosis, leaving the patient with an end colostomy. During Hartmann’s reversal, a colorectal anastomosis was constructed at 6 cm from the anal verge. Formation of a diverting ileostomy was not feasible due to a short mesentery and the presence of prominent panniculus. After 2VSC and a total of 12 days of treatment, the anastomosis was healed.

Patient #13 (prophylactic; female, 32; Crohn’s colitis): A colonic stenosis associated with Crohn’s disease prompted sigmoid resection, as no dysplasia had been detected during prior endoscopic surveillance. Given the patient’s ongoing corticosteroid therapy (prednisolone 40 mg daily), the anastomosis level at 14 cm from the anal verge, and a history of prior ileocolic resection for terminal ileitis, a prophylactic VSC was selected instead of a diverting loop ileostomy. The VSC was dislodged on day 7, with an uneventful postoperative course.

## Discussion

To date, this report represents the largest published experience with the VACStent Colon (VSC). In this series, we present our use of this new device for several indications, anastomotic leakage (AL) being the most frequent, followed by rectal injury and as a prophylactic measure for high-risk colorectal anastomoses. Additionally, we found that the VSC can safely be continued following hospital discharge in selected patients.

Continuous luminal patency without dislocation was achieved in 89.4% of VSCs. Only 5 out of 47 VSCs showed signs of dislocation, indicating a high rate of treatment following the expected course. Most dislocations did not require intervention, as they were detected only during routine endoscopic VSC exchanges and were therefore classified as CDC I. Dislocations requiring earlier-than-planned re-endoscopy were classified as CDC IIIa and occurred only once. Only one complete dislodgement was observed in a prophylactic treatment case on day 7, just hours prior to the scheduled removal.

Notably, none of the observed dislocations occurred during outpatient VSC therapy. We also encountered a case of hemorrhoidal bleeding related to a very distally placed VSC, which required endoscopic management under sedation. The most frequently reported adverse event in our series was pain, particularly with distally positioned VSC, which in some cases necessitated morphine-based analgesia.

Among patients without a diverting loop ileostomy, stool passage remained regular and was supported with macrogol-based stool softeners to reduce the risk of VSC dislodgement by hard faeces.

The severity of AL is typically graded according to the International Study Group of Rectal Cancer (ISREC), which classifies leaks based on the required intervention, largely dictated by clinical symptoms, the presence of sepsis, and overall patient condition [19, 20]. Management options include abdominal lavage with drainage, radiologically guided drainage, diverting stoma formation, resection and creation of an end stoma, and endoscopic vacuum therapy with endosponges, among others [21, 22].

In our experience, the VAC stent is particularly suitable for smaller and superficial defects, for example in the area of an anastomosis. Due to the distal flare of the stent, an ideal distance of several centimeters should be maintained between the defect and the dentate line. The combined use of an endosponge placed into a large presacral cavity with a VSC overlying it, proved particularly effective for treating extensive AL. The VSC helped maintain positioning of the sponge while simultaneously preserving luminal patency, an important advantage over sponge therapy alone.

We also see potential in using the VSC to avoid routine diverting loop ileostomies particularly in low-risk patients who may not require a stoma, as well as in patients who tolerate ileostomies poorly, such as those with impaired renal function. In these situations, the VSC could act as a temporary protective measure, potentially minimizing postoperative complications and reducing the burden associated with stoma formation… The findings of a randomized clinical trial currently underway, which aims to determine if a DLI can be safely omitted altogether, in appropriately selected patients undergoing low anterior resection, have not yet been reported [23]. Prophylactic VSC placement may offer an intermediate approach, reducing both the risk of AL-related complications and the need for a stoma. Although the number of reported prophylactic cases remains limited, prospective studies are warranted to evaluate the impact of VSC on AL incidence in high-risk anastomoses.

While our early experience with the VSC is encouraging, several limitations must be acknowledged. First, this is a single-centre report without a comparative control group. The decision to use the VSC, either therapeutically or prophylactically, was based on clinical judgment rather than predefined inclusion criteria, introducing a potential selection bias. Furthermore, long-term outcomes beyond the study’s follow-up period remain to be assessed, particularly long-term function and cost-effectiveness.

The rate of recorded complications most of which were minor (CDC grade I), should not be interpreted as an argument against the use of VSC per se, but rather reflects the meticulous documentation standards upheld at our institution, which enable the identification and reporting of such events even in a retrospective analysis. However, minor and transient events for example during sedation for endoscopy such as short episodes of hypoxia or hypotension not requiring intervention beyond brief supportive measures may have been underreported due to the retrospective design.

Despite these limitations, our findings suggest that VSC therapy may address a critical gap in colorectal surgery, the ability to promote anastomotic healing by local washout and suction-based sealing without resorting to a diverting ostomy. This may be particularly valuable in younger, low- to intermediate-risk patients, for whom a temporary ileostomy carries significant psychosocial and physical burden. Additionally, the successful implementation of outpatient VSC therapy, as demonstrated in our cohort, which could help mitigate the costs of VSC therapy by reducing length of hospital stay.

In summary, early experiences with the VSC are promising and should promote larger comparative studies to determine the true efficacy and safety of the VSC in both therapeutic and prophylactic settings, and to establish standardised treatment protocols.

## Conclusion

In conclusion, our one-year experience with the VACStent Colon (VSC) demonstrates its potential as both a therapeutic and prophylactic tool in the management of colorectal anastomotic leakage and high-risk anastomoses. The VSC offers a unique combination of vacuum therapy and mechanical support while preserving luminal patency, with promising early outcomes including successful implementation for outpatient use. While complications such as pain and dislocation require careful management the device represents a valuable addition to the armamentarium in selected cases when used within an experienced interdisciplinary setting.

## References

[1] McDermott FD, Heeney A, Kelly ME, Steele RJ, Carlson GL, Winter DC (2015) Systematic review of preoperative, intraoperative and postoperative risk factors for colorectal anastomotic leaks. Br J Surg 102(5):462–479 25703524 10.1002/bjs.9697

[2] Huisman DE, Reudink M, van Rooijen SJ, Bootsma BT, van de Brug T, Stens J et al (2022) Lekcheck: a prospective study to identify perioperative modifiable risk factors for anastomotic leakage in colorectal surgery. Ann Surg 275(1):e189–e197 32511133 10.1097/SLA.0000000000003853 PMC8683256

[3] de Wit A, Bootsma BT, Huisman DE, van Wely B, van Hoogstraten J, Sonneveld DJA et al (2024) Risk factor targeted perioperative care reduces anastomotic leakage after colorectal surgery: the doublecheck study. Ann Surg. 10.1097/SLA.0000000000006442 38989566 10.1097/SLA.0000000000006442 PMC12695192

[4] Arron MNN, Greijdanus NG, Ten Broek RPG, Dekker JWT, van Workum F, van Goor H et al (2021) Trends in risk factors of anastomotic leakage after colorectal cancer surgery (2011–2019): a Dutch population-based study. Colorectal Dis 23(12):3251–3261 34536987 10.1111/codi.15911 PMC9293104

[5] Matthiessen P, Hallbook O, Rutegard J, Simert G, Sjodahl R (2007) Defunctioning stoma reduces symptomatic anastomotic leakage after low anterior resection of the rectum for cancer: a randomized multicenter trial. Ann Surg 246(2):207–214 17667498 10.1097/SLA.0b013e3180603024 PMC1933561

[6] Degiuli M, Elmore U, De Luca R, De Nardi P, Tomatis M, Biondi A et al (2022) Risk factors for anastomotic leakage after anterior resection for rectal cancer (RALAR study): a nationwide retrospective study of the Italian Society of Surgical Oncology Colorectal Cancer Network Collaborative Group. Colorectal Dis 24(3):264–276 34816571 10.1111/codi.15997 PMC9300066

[7] Niu L, Wang J, Zhang P, Zhao X (2020) Protective ileostomy does not prevent anastomotic leakage after anterior resection of rectal cancer. J Int Med Res 48(8):300060520946520 32862745 10.1177/0300060520946520 PMC7457655

[8] Mu Y, Zhao L, He H, Zhao H, Li J (2021) The efficacy of ileostomy after laparoscopic rectal cancer surgery: a meta-analysis. World J Surg Oncol 19(1):318 34732226 10.1186/s12957-021-02432-x PMC8567543

[9] Tsujinaka S, Suzuki H, Miura T, Sato Y, Shibata C (2022) Obstructive and secretory complications of diverting ileostomy. World J Gastroenterol 28(47):6732–6742 36620340 10.3748/wjg.v28.i47.6732 PMC9813931

[10] Talboom K, Vogel I, Blok RD, Roodbeen SX, Ponsioen CY, Bemelman WA et al (2021) Highly selective diversion with proactive leakage management after low anterior resection for rectal cancer. Br J Surg 108(6):609–612 33793724 10.1093/bjs/znab018

[11] Denost Q, Sylla D, Fleming C, Maillou-Martinaud H, Preaubert-Hayes N, Benard A (2023) A phase III randomized trial evaluating the quality of life impact of a tailored versus systematic use of defunctioning ileostomy following total mesorectal excision for rectal cancer-GRECCAR 17 trial protocol. Colorectal Dis 25(3):443–452 36413078 10.1111/codi.16428

[12] Holmgren K, Haggstrom J, Haapamaki MM, Matthiessen P, Rutegard J, Rutegard M (2021) Defunctioning stomas may reduce chances of a stoma-free outcome after anterior resection for rectal cancer. Colorectal Dis 23(11):2859–2869 34310840 10.1111/codi.15836

[13] Yohannes A, Knievel J, Lange J, Dormann AJ, Hugle U, Eisenberger CF et al (2024) VacStent as an innovative approach in the treatment of anastomotic insufficiencies and leakages in the gastrointestinal tract-review and outlook. Life. 10.3390/life14070821 39063574 10.3390/life14070821 PMC11277949

[14] Lange J, Knievel J, Wichmann D, Kahler G, Wiedbrauck F, Hellmich T et al (2023) Clinical implantation of 92 VACStents in the upper gastrointestinal tract of 50 patients-applicability and safety analysis of an innovative endoscopic concept. Front Surg 10:1182094 37215348 10.3389/fsurg.2023.1182094 PMC10198570

[15] Dell’Anna G, Fanti L, Fanizza J, Bara R, Barchi A, Fasulo E et al (2024) VAC-stent in the treatment of post-esophagectomy anastomotic leaks: a new, “kid on the block” who marries the best of old techniques-a review. J Clin Med. 10.3390/jcm13133805 38999371 10.3390/jcm13133805 PMC11242239

[16] Heiss MM, Lange J, Knievel J, Yohannes A, Hugle U, Dormann AJ et al (2024) Treatment of anastomotic leak in colorectal surgery by endoluminal vacuum therapy with the VACStent avoiding a stoma - a pilot study. Langenbecks Arch Surg 409(1):234 39083099 10.1007/s00423-024-03426-5 PMC11291571

[17] Herner A, Nennstiel S, Ramser M, Turina M, Schlag C (2025) New therapeutic approach for anastomotic leaks after ileoanal J-pouch construction in patients with ulcerative colitis. Gastrointest Endosc 101(1):222–223 39265742 10.1016/j.gie.2024.09.004

[18] Clavien PA, Strasberg SM (2009) Severity grading of surgical complications. Ann Surg 250(2):197–198 19638901 10.1097/SLA.0b013e3181b6dcab

[19] Rahbari NN, Weitz J, Hohenberger W, Heald RJ, Moran B, Ulrich A et al (2010) Definition and grading of anastomotic leakage following anterior resection of the rectum: a proposal by the International Study Group of Rectal Cancer. Surgery 147(3):339–351 20004450 10.1016/j.surg.2009.10.012

[20] Heuvelings DJI, Mollema O, van Kuijk SMJ, Kimman ML, Boutros M, Francis N et al (2024) Quality of reporting on anastomotic leaks in colorectal cancer trials: a systematic review. Dis Colon Rectum 67(11):1383–1401 39111814 10.1097/DCR.0000000000003475 PMC11477855

[21] Chiarello MM, Fransvea P, Cariati M, Adams NJ, Bianchi V, Brisinda G (2022) Anastomotic leakage in colorectal cancer surgery. Surg Oncol 40:101708 35092916 10.1016/j.suronc.2022.101708

[22] Tsai YY, Chen WT (2019) Management of anastomotic leakage after rectal surgery: a review article. J Gastrointest Oncol 10(6):1229–1237 31949944 10.21037/jgo.2019.07.07 PMC6955017

[23] Rutegard M, Lindskold M, Jorgren F, Landerholm K, Matthiessen P, Forsmo HM et al (2025) SELective defunctioning stoma approach in low anterior resection for rectal cancer (SELSA): protocol for a prospective study with a nested randomized clinical trial investigating stoma-free survival without major LARS following total mesorectal excision. Colorectal Dis 27(2):e70009 39887540 10.1111/codi.70009 PMC11780343

